# Fused Membrane-Targeted Nanoscale Gene Delivery System Based on an Asymmetric Membrane Structure for Ischemic Stroke

**DOI:** 10.3390/pharmaceutics17101357

**Published:** 2025-10-21

**Authors:** Jing Shi, Xinyi Zhao, Yue Zhang, Zitong Zhao, Jing Wang, Jia Mi, Zhaowei Xu, Chunhua Yang, Jing Qin, Hong Zhang

**Affiliations:** 1 Department of Pharmaceutics, School of Pharmaceutical Sciences, Fudan University & Key Laboratory of Smart Drug Delivery, Ministry of Education & State Key Laboratory of Advanced Drug Formulations for Overcoming Delivery Barriers, Shanghai 201203, China; 21211030026@m.fudan.edu.cn (J.S.); 22211030048@m.fudan.edu.cn (X.Z.); 19211030064@fudan.edu.cn (Y.Z.); 23211030111@m.fudan.edu.cn (Z.Z.); 2 Zhejiang Cancer Hospital, Hangzhou Institute of Medicine (HIM), Chinese Academy of Sciences, Hangzhou 310022, China; wangjing5678@zju.edu.cn; 3 Department of Nuclear Medicine and PET Center, The Second Affiliated Hospital of Zhejiang University School of Medicine, Hangzhou 310009, China; 4 Institute of Nuclear Medicine and Molecular Imaging, Zhejiang University, Hangzhou 310058, China; 5 Key Laboratory of Medical Molecular Imaging of Zhejiang Province, Hangzhou 310009, China; 6 Precision Medicine Research Center, School of Pharmacy, Binzhou Medical University, Yantai 264003, China; jia.mi@bzmc.edu.cn (J.M.); zhaoweixv@bzmc.edu.cn (Z.X.); yangchunhua@bzmc.edu.cn (C.Y.); 7 Key Laboratory for Biomedical Engineering of Ministry of Education, Zhejiang University, Hangzhou 310027, China; 8 College of Biomedical Engineering and Instrument Science, Zhejiang University, Hangzhou 310058, China

**Keywords:** exosome, ischemic stroke, lipid calcium phosphate, nanoparticle, brain-targeted delivery, fused membrane

## Abstract

**Background**: Bone marrow-derived mesenchymal stem cell exosomes (EXOs) are attractive in biotechnology and biomedical research, as they possess natural cell-targeting properties and can cross biological barriers by influencing the SDF-1/CXCR4 axis. Lipid calcium phosphate (LCP) consists of a calcium phosphate core and an asymmetric phospholipid bilayer containing abundant Ca^2+^ ions. AMD3100 modification of targeted LCP (T-LCP) can achieve targeted delivery to ischemic lesions via specific binding to CXCR4 receptors on various neuronal cell surfaces. **Methods**: Herein, a fused membrane formulation that simultaneously possesses EXO characteristics and enables targeted modification with AMD3100 was produced. The characteristics of biologically derived EXOs, artificially designed T-LCP, and the fused membrane formulation, including targeted delivery and gene loading efficiency, were then compared. **Results**: The fusion of artificially designed T-LCP with EXOs of natural origin is feasible and combines the advantages of both to achieve more prominent targeted delivery effects. **Conclusions**: MiRNA210-based gene therapy was effective in this study and provides a strategy for therapeutic efficacy in delivery systems with different targeting efficiencies.

## 1. Introduction

Stroke is the leading cause of disability and the second leading cause of death worldwide. One of main bottlenecks for the treatment of ischemic stroke (IS) lies in the unsatisfactory efficacy of administering therapeutic agents. Researchers have engineered diverse nanoparticle-based drug delivery systems to improve the therapeutic outcomes of IS [1]. Various nanoparticles allow for specific designs to meet different therapeutic goals or modifications with various ligands and responsive linkers for intelligent drug delivery [2]. Engineered exosomes (EXOs) for drug delivery have also garnered considerable attention [3,4]. Bone marrow-derived mesenchymal stem cell EXOs possess immense potential in the treatment of IS. By influencing the SDF-1/CXCR4 axis, they are capable of targeting the ischemic brain region to promote post-ischemic neurological and vascular repair [5]. EXOs are highly active, are stable in body fluids, have low immunogenicity, and possess the natural targeting properties of cells and the ability to cross biological barriers [6]. However, their low production and isolation rates, insufficient purity, low drug-carrying capacity, high engineering costs, and potential safety issues, such as complex heterogeneity, substantially limit their clinical translation [7,8]. Alternatively, artificially designed nanoparticles (NPs) can be developed with targeting moieties for specific receptors or efficiently loaded with gene drugs to fully utilize their advantages in a highly controllable manner. However, an issue in the design of targeting moieties is whether physiological activity can be fully retained. Therefore, the development of methods to obtain simultaneously efficient and controllable gene-targeted delivery systems is a key aim in the field of pharmacy [9].

Lipid calcium phosphate (LCP) consists of a calcium phosphate (CaP) core and an asymmetric phospholipid bilayer enriched with Ca^2+^ ions. Its positive charge allows a high gene-loading efficiency and creates a “proton sponge” effect, thereby realizing the efficient intracellular delivery and release of genes. LCP is a novel delivery system that has attracted substantial attention in the field [10]. AMD3100 is an antagonist of the CXCR4 receptor. NP surfaces have been modified with AMD3100 to allow for specific binding to CXCR4 receptors on the surfaces of a variety of neuronal cells and achieve targeted delivery to ischemic lesions. The underlying mechanisms of such AMD3100-based targeted delivery systems are similar to those of biologically derived EXOs [5]. The SDF-1/CXCR4 axis, for example, plays an important role in the construction of targeted delivery systems [11,12].

Biologically derived EXOs are involved in a variety of physiological processes, contain specific proteins and genes of cellular origin, and possess an inherent homing ability and strong bioactivity [13]. Artificially designed NPs can be efficiently modified and loaded for the delivery of dominant targeting moieties and target miRNAs [14]. Therefore, we compared the characteristics and advantages of natural EXOs and artificially designed NPs. We also aimed to utilize the features of unsynchronized inner and outer phospholipid layer formation during the LCP preparation process to combine EXOs with the outer phospholipid layer, thereby obtaining a combined membrane formulation that fused EXO characteristics and enables targeted modification with AMD3100. The characteristics of biologically derived EXOs, artificially designed NPs, and fused membrane formulation, including targeted delivery and gene loading efficiency, were then compared.

Although some progress has been made in developing effective treatments for IS, there is still a need for the development of therapeutic agents for neuroprotection. EXOs have recently emerged as ideal therapeutic candidates for IS because of their ability to pass through the blood–brain barrier and mediate intercellular communication [15]. It has been reported that EXOs derived from miRNA-210-overexpressing bone marrow mesenchymal stem cells (BMSCs) can protect against lipopolysaccharide injury [16]. The enhanced miRNA-210 expression has also been found to promote cerebral protection following ischemic brain injury [17]. Thus, we loaded miRNA-210, which is involved in angiogenesis, neurogenesis, and neural precursor cell migration [18], into LCP and the fused membrane formulation. Considering that miRNA-210-containing BMSC-derived EXOs have been reported in the literature, we assessed the in vitro gene-transfection efficiency, in vivo targeted delivery efficiency, and beneficial effects of AMD3100-modified targeted LCP (T-LCP), EXOs, and the fused membrane formulation on neurological function in an animal model of IS. The characteristics of nanoscale formulations and biologically derived EXOs, as well as the possibility of combining the advantages of NPs and EXOs, were evaluated. This study clarifies the characteristics of different formulations in targeted delivery and provides an efficient, stable delivery system as a promising strategy for gene therapy in IS.

## 2. Materials and Methods

### 2.1. Materials

1,2-Dioleoyl-3-trimethylammonium-propane (DOTAP) chloride salt and dioleoylphosphatydic acid (DOPA) were purchased from Avanti Polar Lipids Inc. (Alabaster, AL, USA); DSPE-PEG2000-NHS and DSPE-PEG2000 were purchased from A.V.T. Pharmaceutical Co., Ltd. (Shanghai, China); Cy5-miRNA-210, miRNA210-Green fluorescent protein (miRNA210-GFP), and miRNA-Negative were purchased from GenePharma Co., Ltd. (Shanghai, China); Igepal CO-520 was purchased from Sigma-Aldrich (St. Louis, MO, USA); Lipo2000-GFP and α-MEM medium with GlutaMAX™-I were obtained from Thermo Fisher Scientific (Waltham, MA, USA); TSG101, HSP70, GM130, and Goat anti-rabbit IgG (H + L)-HRP antibodies, as well as LysoTracker Green, were purchased from Beyotime (Shanghai, China); and DiD, Dil, DiO, DMSO, Triton X-100, and DEPC water were purchased from Dalian Meilun Biotechnology Co., Ltd. (Dalian, China). BMSC and PC12 (highly differentiated neuronal cells) were purchased from the Cell Bank/Stem Cell Bank of the Chinese Academy of Sciences (Beijing, China). C57 mice (male, 23 ± 2 g) were obtained from the Animal Experiment Center of Fudan University. All animal experiments were performed in accordance with the ethical guidelines approved by the Institutional Animal Care and Use Committee of Fudan University (no. 2020-04-YJ-QJ-01; Shanghai, China).

### 2.2. Extraction and Characterization of EXOs

EXOs were extracted by ultracentrifugation [18]. BMSCs in the logarithmic growth phase were obtained and washed thrice with 1× phosphate-buffered saline (PBS). Serum-free culture medium was added, the cells were cultured for another 48 h, and the cell supernatant was collected at 4 °C. Centrifugation was performed at 500× *g* for 5 min, and the resultant supernatant was collected at 4 °C. Re-centrifugation was performed for the collected supernatant at 2000× *g* for 30 min, and the resultant supernatant was collected at 4 °C. Re-centrifugation was then performed for the collected supernatant at 10,000× *g* for 60 min, and the resultant supernatant was collected at 4 °C. Re-centrifugation was further performed on the collected supernatant at 120,000× *g* for 70 min, and the precipitate obtained after the supernatant had been discarded was resuspended in sterile 1× PBS. The obtained EXOs were diluted with purified water to an appropriate concentration. Particle size was determined using a NanoSight nanoparticle tracking analysis (NTA) system (Malvern Panalytical, Malvern, UK); zeta potential was measured using a Malvern dynamic light scattering (DLS) system (Malvern); morphology was observed under a transmission electron microscope (TEM) (FEI, USA); and protein markers were evaluated using Western blotting (Bio-Rad, Fort Worth, TX, USA).

### 2.3. Establishment of a Middle Cerebral Artery Occlusion (MCAO) Model

The middle cerebral artery is a stroke-prone site. Therefore, the MCAO model has become a widely recognized standard animal model of focal cerebral ischemia and exhibits clinical symptoms similar to those of cerebral ischemia [19]. C57 mice weighing 23 ± 2 g were fasted for 12 h preoperatively and allowed ad libitum access to drinking water. Each mouse was weighed, and body weight was recorded. The mice were anesthetized using intraperitoneal injections of 10% chloral hydrate solution and fixed in the supine position after anesthesia. Each mouse was cut open along the middle of the neck, and the common carotid artery (CCA) was separated. Subsequently, the point where the CCA bifurcates into the external carotid artery (ECA) and internal carotid artery (ICA) was located towards its distal end. The ECA was separated in the middle, and two 6-0 sutures were passed through it. The sutures were knotted tightly at the distal end and clamped with a hemostat at the proximal end. In between the two knots on the ECA, a small incision was carefully made using ophthalmic scissors, and an appropriately sized nylon filament was gently inserted into the ECA. After determining that the suture had been successfully inserted into the artery, the filament was redirected into the ICA and advanced until resistance was met. At this point, the middle cerebral artery was blocked by the filament, causing local ischemia for 90 min. The diameter of the filament was 0.16 ± 0.01 mm, and the diameter of the head silicone bolt was 0.2 ± 0.01 mm. The 6-0 sutures at the proximal end of the ECA were knotted tightly to secure the filament, and the mouse was placed on a heating pad to maintain its body temperature. After 90 min, the filament was removed to complete the establishment of the mouse model of ischemia–reperfusion. After animal modeling, mice with a score of 2–4 were selected for subsequent experiments according to the Longa scale. The mice in the sham operation (sham) group were only subjected to CCA without subsequent surgery.

### 2.4. Preparation of LCP/T-LCP

To prepare the CaP core [14], a reverse microemulsion method was adopted. Two 20 mL portions of a mixture of cyclohexane and the surfactant Igepal CO-520 (71:29, *v*/*v*) were separately added to two 100 mL eggplant-shaped flasks. Then, 300 μL of CaCl_2_ solution (2.5 M) was added dropwise to one portion, and 300 μL of Na_2_HPO_4_ solution (12.5 mM) was added to the other portion. The two portions were separately mixed uniformly via magnetic stirring for 30 min for the preparation of the Ca^2+^ and HPO_4_^2−^ phases. Then, 150 μL of a chloroform solution of DOPA (20 mM) was added dropwise to the HPO_4_^2−^ phase, and stirring was continued for 30 min. The Ca^2+^ and HPO_4_^2−^ phases were combined and mixed uniformly via magnetic stirring for 30 min. After the addition of 40 mL of anhydrous ethanol, the mixture was centrifuged at 12,000× *g* for 20 min at 4 °C. After the supernatant had been discarded, 20 mL of anhydrous ethanol was added. The mixture was mixed uniformly through vortexing, and centrifugation was performed twice. After the evaporation of the anhydrous ethanol, the precipitate was resuspended in a chloroform solution to obtain the CaP core, which was stored at −20 °C before further use.

**Scheme 1:**
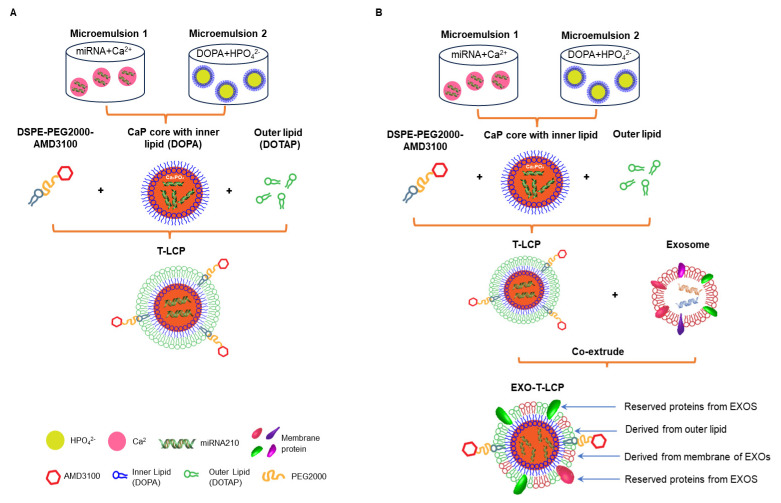
The preparation process of T-LCP (**A**) and EXO-T-LCP (**B**).

To prepare LCP/T-LCP, DOTAP, cholesterol, and DSPE-PEG2000 (6 mol%) with a total molar mass of 50 mmol were weighed and added to a 10 mL centrifuge tube. The mixture was dissolved with 5 mL of chloroform and mixed with the CaP core-containing chloroform solution. After the addition of 90 μL of DiD in chloroform solution (2 mg/mL), the mixture was mixed uniformly in a water bath at 25 °C, and the organic reagents were removed via vacuum rotary evaporation for 20 min. This led to the formation of a uniform film on the walls of the eggplant-shaped flask. Next, 3 mL of PBS was added to the flask, and hydration was performed for 2 h. LCP was obtained by collecting the intact particles that remained after the samples had been sequentially passed through 400, 200, and 100 nm polycarbonate membranes. Particle size and zeta potential were determined using a DLS system. The LCP was then stored in a refrigerator at 4 °C for further use. DSPE-PEG2000-AMD3100 (2.4‰ of the total molar mass) was added to the phospholipid formulation, dissolved with 5 mL of chloroform, and subjected to the preparation method described above to obtain the modified targeted LCP (T-LCP, Scheme 1).

To prepare miRNA-loaded T-LCP, Cy5-miRNA-210 or GFP-miRNA-210 dry powder was centrifuged at 3500 rpm for 1 min, dissolved in 125 μL of DEPC-treated water, mixed with 300 μL of CaCl_2_ solution (2.5 M), added to the oil phase, and mixed to form the Ca^2+^ phase. Subsequently, T-LCP@miR was obtained using the method described above. Scrambled miRNAs were used as a negative control (T-LCP@NC) for pharmacodynamic assessments.

### 2.5. Preparation of Fused Membrane Formulations and Determination of the Optimal Fusion Ratio

EXOs and LCP were mixed in different mass ratios (1:1, 1:4, and 1:9) and repeatedly co-extruded through a 200 nm polycarbonate membrane several times to obtain the fused membrane formulation EXO-LCP. Particle size and zeta potential were determined using NTA system or DLS system. The protein markers of EXO-LCP were evaluated using Western blotting. EXO-LCP and a physical mixture of EXOs and LCP (EXO + LCP) were characterized by fluorescence correlation spectroscopy (FCS) to examine the state of fusion. EXO-LCP was then dropped onto a copper mesh and air-dried. After negative staining with 2% phosphotungstic acid, EXO-LCP was observed using TEM.

EXO-LCP formulations with different ratios were co-incubated with PC12 cells in a 37 °C incubator for 2 h, after which the cellular uptake of EXO-LCP was measured using flow cytometry. EXO-LCP samples at different ratios were diluted with purified water, and the particle size, polydispersity index (PDI), and zeta potential were measured using the Malvern DLS system. The fused formulation EXO-T-LCP was prepared from T-LCP and EXOs using the method described above.

### 2.6. Uptake of Different Formulations in Two Types of Cells

PC12 and BCEC cells were separately inoculated into 12-well plates (2 × 10^5^ cells/mL) and cultured for 24 h. The different formulations (PBS, LCP@DiD, T-LCP@DiD, EXO-LCP@DiD, EXO-T-LCP@DiD, and EXO@DiD) were separately added (30 μL/mL) and co-incubated with the cells for 0.5 or 2 h. Subsequently, the cells were washed thrice with 1× PBS, fixed with 4% paraformaldehyde at 37 °C for 15 min, washed thrice with 1× PBS, treated with 250 μL of DAPI working solution per well for 5 min, washed thrice with 1× PBS, mounted with an anti-fade agent, and subjected to fluorescence observations under a confocal microscope.

### 2.7. Lysosomal Escape of Different Formulations in Two Cell Types

PC12 and BCEC cells were separately inoculated into confocal Petri dishes (1 × 10^5^ cells/mL) and cultured overnight. The cells were washed thrice with 1× PBS 8 and 12 h after adding the different formulations (LCP@DiD, T-LCP@DiD, EXO-LCP@DiD, EXO-T-LCP@DiD, and EXO@DiD). Next, 1 mL of LysoTracker Green was added to each well, and the cells were incubated at 37 °C for 30 min and washed thrice with 1× PBS. After the addition of 200 μL of Hoechst 33342 working solution to each well, the cells were further incubated at 37 °C for 20 min. After incubation, the cells were washed thrice with 1× PBS, supplemented with 1 mL of culture medium, and placed under a spinning disk confocal microscope to observe the distribution of each formulation in lysosomes. The semi-quantitative statistical analysis was made for the confocal images after 2 h incubation.

### 2.8. Transfection Efficiency of Green Fluorescent Protein (GFP)-Loaded Formulations in Different Cells

To prepare the transfection reagent in accordance with the number of cells used for inoculation, Lipofectamine 2000 and the GFP-miRNA-210 plasmid were diluted to a certain concentration with culture medium. The diluted Lipofectamine 2000 and plasmid solutions were mixed (1:1, *v*/*v*) and incubated for 5 min to obtain the GFP transfection reagent, which was used immediately after preparation.

PC12 and BCEC cells were separately inoculated into 12-well plates (1 × 10^5^ cells/mL) and co-incubated with the formulations (PBS, LCP@GFP, T-LCP@GFP, EXO-LCP@GFP, EXO-T-LCP@GFP, and Lipo2000@GFP) for 48 h. Subsequently, the cells were washed thrice with 1× PBS, fixed with 4% paraformaldehyde solution at 37 °C for 15 min, washed thrice with 1× PBS, treated with 250 μL of DAPI working solution for 5 min, and washed thrice with 1× PBS before being mounted with an anti-fade agent. GFP expression was observed under a confocal microscope.

### 2.9. Protective Effects of miRNA-210-Loaded Formulations Against Mitochondrial Damage in Different Cells

PC12 and BCEC cells were inoculated onto 12-well plates (2 × 10^5^ cells/mL) and incubated overnight. After incubation in culture medium containing CoCl_2_ for 30 min, the different formulations (PBS, LCP@miR, T-LCP@miR, EXO-LCP@miR, EXO-T-LCP@miR, EXO, and T-LCP@NC) were added, and incubation was continued for 48 h. Cells without the added CoCl_2_ served as the normal control (normal) group. Following incubation, 100 μL of trypsin was added to each well for cell digestion at 37 °C for 2 min, after which 400 μL of culture medium was added to terminate digestion. The cell suspension was transferred to a 1.5 mL centrifuge tube and centrifuged at 1000 rpm for 5 min. After the supernatant had been discarded, the cells were resuspended in 500 μL of culture medium, and 500 μL of JC-1 dye working solution was added for incubation at 37 °C for 2 min. Centrifugation was performed at 600× *g* at 4 °C for 4 min, and the supernatant was discarded. After being washed twice with 500 μL of 1× JC-1 working solution, the contents of each tube were resuspended in 500 μL of 1× JC-1 working solution. Mitochondrial membrane potential was measured using flow cytometry to evaluate the protective effects of the different formulations against mitochondrial damage.

### 2.10. Assessment of the In Vivo Targeting of Different Formulations

The mice were divided into four groups (n = 3 per group). Twelve and 24 h after the model had been established, the formulations (T-LCP@DiD, EXO-T-LCP@DiD, EXO@DiD, or an equivalent volume of PBS) were separately injected into the tail vein of each mouse at a dose of 100 µg/kg (based on DiD). The mice were anesthetized 1, 2, 4, 8, 12, and 24 h after the second drug administration and placed in a small-animal live imaging system for imaging (Ex = 640 nm, Em = 680 nm). Cardiac perfusion was performed, and the brain, heart, liver, spleen, lungs, and kidney tissues were collected for ex vivo tissue imaging.

### 2.11. Pharmacodynamic Assessment of EXO-LCP-Fused Membrane Formulations

The mice were subjected to behavioral assessments in accordance with the modified neurological severity score (mNSS) criteria [20]. Scores ranged from 0–18 points, with the normal mice having a score of 0, 1–6 points indicating mild injury, 7–12 points indicating moderate injury, and 13–18 points indicating severe injury [21]. The mice were randomly divided into six groups: sham, PBS, T-LCP@NC (negative control), EXO, T-LCP@miR, and EXO-T-LCP@miR. Each group consisted of six mice subjected to tail vein injections of the respective formulations 12 and 24 h after model establishment. mNSS scoring was performed for the behavioral assessments 24 and 48 h after the second drug administration.

## 3. Results and Discussion

### 3.1. Characterization of EXO-LCP-Fused Membrane Formulations and Determination of the Optimal Fusion Ratio

**Figure 1:**
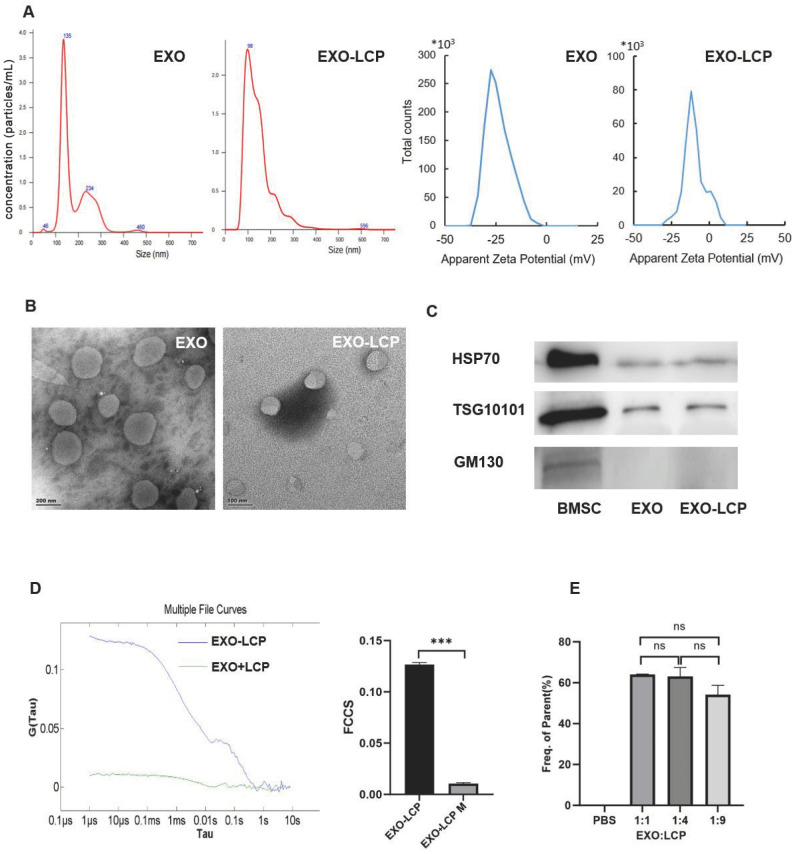
The confirmation and characteristics of fusion preparations. (**A**): The size distribution and zeta potential profile of EXO and EXO-LCP; (**B**): The TEM photos of EXO and EXO-LCP. (**C**): The membrane protein markers of BMSC, EXO and EXO-LCP in Western blotting assay. (**D**): The spectrum of fluorescence correlation spectroscopy and its quantitative results. (**E**): The uptake of EXO-LCP prepared with different ratios of EXO:LCP in PC12 cells. ^ns^ *p* > 0.05, *** *p* < 0.001.

A diameter of approximately 135 nm and a potential of −25.8 mV were determined for the EXOs, while 98 nm and −9.71 mV were determined for EXO-LCP (Figure 1A). The quasi-circular shape was further observed under a TEM (Figure 1B). Western blotting results shown in Figure 1C indicate that EXO-LCP contained the protein markers HSP70 and TSG101, homologous to BMSCs, but did not contain the cellular marker GM130. These findings indicated the successful fusion of EXOs to the outer lipid layer of LCP. FCS (Figure 1D) showed a significant correlation between the physically mixed EXO + LCP sample and EXO-LCP, demonstrating the successful preparation of the fused NPs. The morphological characterization of EXO-LCP through TEM revealed the presence of intact, round particles, with diameters of approximately 100 nm (Figure 1B).

EXO-LCP prepared in different ratios showed no significant differences in uptake by PC12 cells (*p* = 0.9867 for 1:1 vs. 1:4; *p* = 0.0579 for 1:1 vs. 1:9; *p* = 0.0571 for 1:4 vs. 1:9); however, a slight decline was observed in EXO-LCP at a ratio of 1:9 (Figure 1E). As shown in Table 1, an increase in the LCP proportion reduced the particle size, with no significant difference in PDI. This demonstrated that the fusion of LCP with EXOs produced formulations with relatively stable and uniform particle sizes. The smaller particle size of LCP may explain the decrease in particle size of the fused formulations. Given the positive potential of LCP and negative potential of EXOs, increasing the ratio of EXOs reduced formulation charge from positive to negative, with a gradual decrease as the EXO ratio increased. Based on these findings, an LCP:EXO ratio of 1:4 was adopted for the preparation of fused membrane formulations. LCP was first reported by Jun Li in 2012 [10] and comprised CaP NPs with an asymmetric lipid bilayer made via the microemulsion method. The hydrophobic CaP core coated with DOPA was also determined to provide a variety of choices for the outer leaflet lipid in the bilayer surface to make a water-soluble NP [22], such as a fused membrane in studies on nonviral gene therapies.

### 3.2. Uptake of Fused Membrane Formulations in Different Cells and Their Relevant Mechanisms

**Figure 2:**
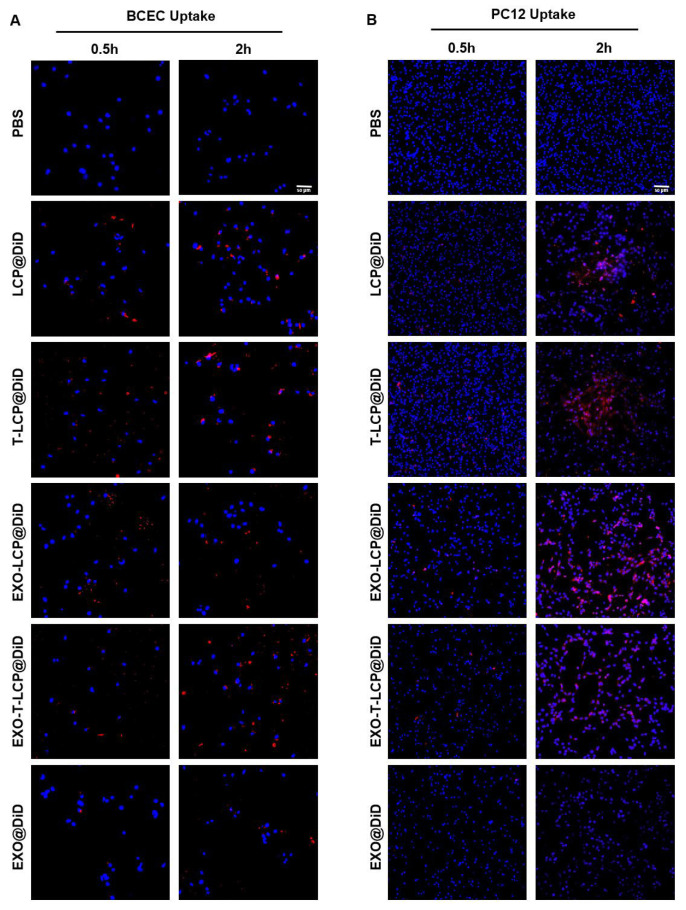

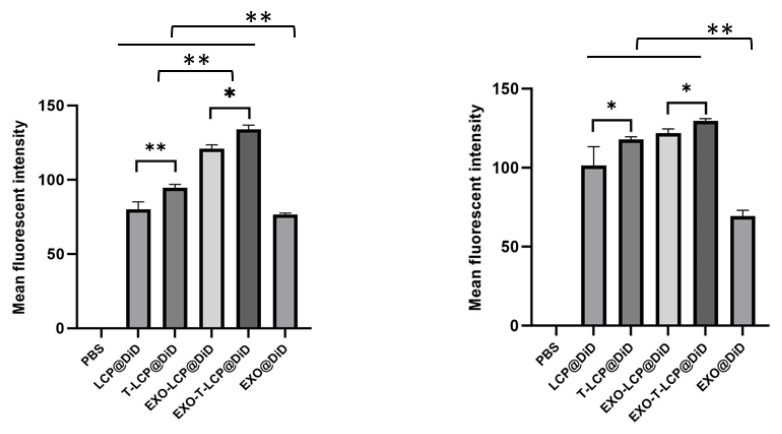
Intracellular uptake of different preparations in different cells. (**A**): Intracellular uptake of different preparations in BCEC cell and its semi-quantitative results after 2 h incubation; (**B**): Intracellular uptake of different preparations in PC12 cell and its semi-quantitative results after 2 h incubation. Red: DiD, Blue: DAPI, Scale bar = 50 μm. * *p* < 0.05, ** *p* < 0.01.

The uptake of the different formulations during incubation with BCEC or PC12 cells increased significantly over time, with significantly higher uptake after 2 h than after 30 min (Figure 2A,B). After the different formulations had been co-incubated 2 h separately with BCEC cells, T-LCP uptake was found to be significantly higher than LCP uptake (*p* = 0.0039). Uptake was higher for both the EXO-T-LCP and EXO-LCP fused membrane formulations than for the corresponding unfused T-LCP, LCP, and EXO formulations. Between the two types of fused membrane formulations, EXO-T-LCP showed significantly higher uptake than EXO-LCP (*p* = 0.0254). Therefore, among the different formulations, EXO-T-LCP showed the highest uptake in BCEC cells.

The uptake of the various formulations by PC12 cells was similar to that by BCEC cells, with a greater uptake of the modified targeted formulations than of normal LCP and greater uptake of fused membrane formulations than of unfused formulations. EXO-T-LCP exhibited significantly higher uptake than EXO-LCP (*p* = 0.021). Notably, all the formulations had higher cellular uptake in BCEC cells than in PC12 cells during the first 30 min of incubation; however, uptake was higher in PC12 cells than in BCEC cells after 2 h of incubation. Previous research has shown that the CXCR4 receptor is highly expressed on the surfaces of PC12 cells, and this target is associated with the repair of PC12 cell damage [23].

### 3.3. Lysosomal Escape of Fused Membrane Formulations in Cells

**Figure 3:**
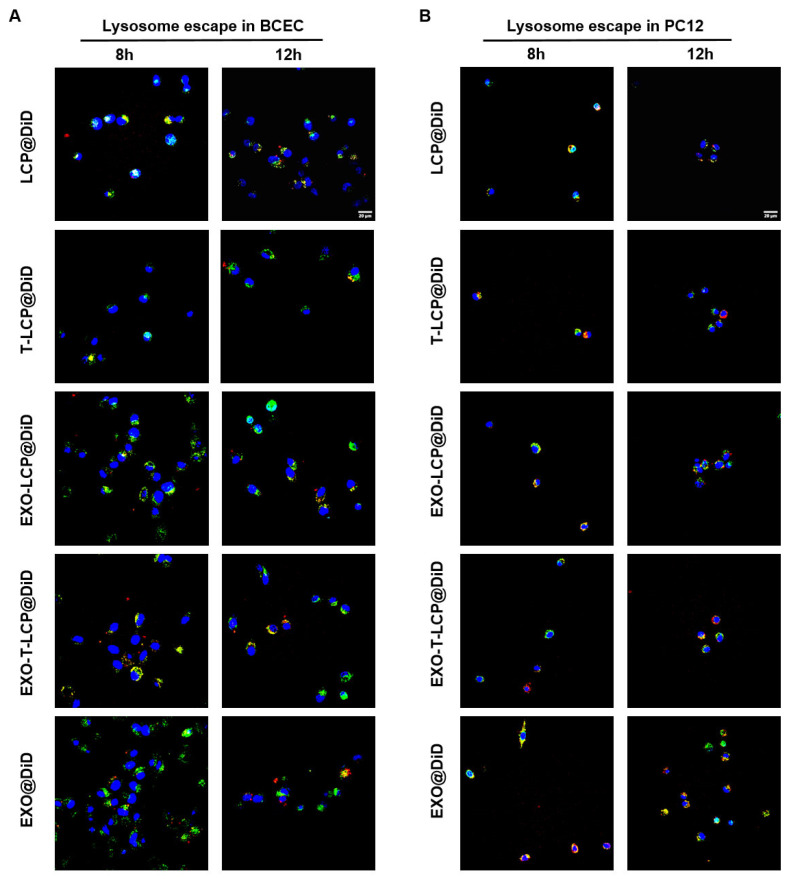
Lysosomal escape of different preparations in different cells within 12 h. (**A**): BCEC cells; (**B**): PC12 cells. Red: DiD, Green: LysoTracker, Blue: Hoechst 33342, Scale bar = 20 μm.

Lysosomal escape of unfused LCP and T-LCP was insignificant after 8 h (Figure 3A,B). However, a clear separation of the fluorescence signals for the two formulations from the lysosomal signals was observed after 12 h, demonstrating that lysosomal escape was possible after a certain amount of time. For the EXO formulation, separation from the lysosomal signals could be seen after 8 h, and an increase in the number of separated fluorescence signals was observed after 12 h. This indicated that the EXO formulation was capable of faster and more persistent lysosomal escape compared with that of the two NP formulations. In the fused membrane formulations, clear signal separation was observed after 8 h, and the signal intensity of the EXO-T-LCP group was higher than that of the EXO-LCP group. After 12 h, the persistent separation of the formulation and lysosomal fluorescence signals was observed in both the EXO-T-LCP and EXO-LCP groups. This indicates that the fused membrane formulations possessed better lysosomal escape capacities. The greater lysosomal escape of T-LCP than of LCP may not be solely determined by the large number of Ca^2+^ ions in LCP but may also be related to the higher uptake of T-LCP, facilitating the observation of lysosomal escape. The fusion of EXOs with LCP notably increased the lysosomal escape capacity of both types of formulations.

In PC12 cells, a clear separation of the formulation and lysosomal fluorescence signals was observed after 8 h in the T-LCP group but not in the LCP group. At 12 h, clear signal separation was seen in both groups, with a higher signal intensity in the T-LCP group than in the LCP group. These results demonstrated that T-LCP was capable of faster and persistent lysosomal escape. For the fused formulations, lysosomal separation was not evident in PC12 cells after 8 h in the EXO-LCP group, while more obvious in the EXO-T-LCP group. At 12 h, both groups exhibited a greater extent of lysosomal escape. Significant signal separation was observed in the EXO group between 8–12 h of incubation, suggesting that the EXOs are capable of more rapid and persistent lysosomal escape in large numbers. Therefore, T-LCP exhibited more effective lysosomal escape than did LCP, and the EXOs demonstrated a stronger escape capacity. The fusion of EXOs with LCPs further enhanced the lysosomal escape capacity of the NP formulations.

### 3.4. Transfection Efficiency of GFP-Loaded Fused Membrane Formulations in Different Cells

**Figure 4:**
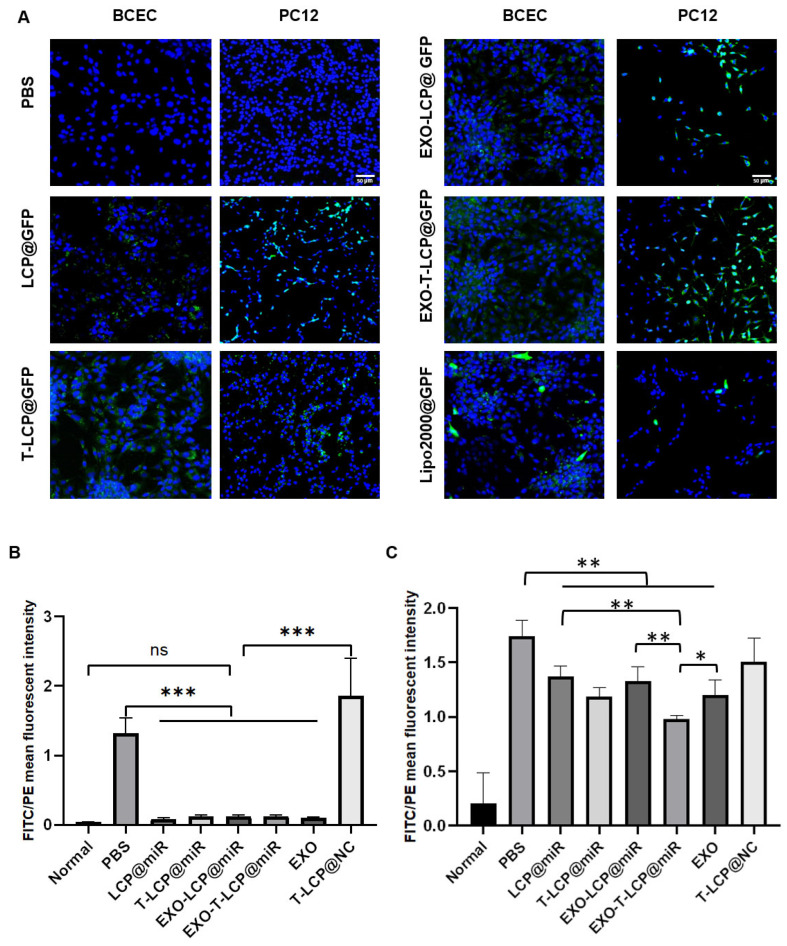
The in vitro activity of different nanoparticles in BCEC and PC12. (**A**): The transfection efficiency of different nanoparticles. Green: GFP protein, Blue: DAPI, Scale bar = 50 μm. (**B**): The antioxidant activity efficiency of different nanoparticles in BCEC. (**C**): The antioxidant activity efficiency of different nanoparticles in PC12. ^ns^ *p* > 0.05, * *p* < 0.05, ** *p* < 0.01, *** *p* < 0.001.

Figure 4A shows the transfection efficiencies of different GFP-loaded formulations in BCEC and PC12 cells. In BCEC cells, T-LCP showed better transfection efficiency than that of LCP. Fusion with EXOs improved the transfection efficiency for both formulations, with the best results obtained for EXO-T-LCP. Similar results were observed in PC12 cells. Therefore, it can be deduced that the EXO–membrane fusion contributed to an increase in transfection efficiency.

### 3.5. Assessment of the In Vitro Antioxidant Activity of Fused Membrane Formulations

To investigate the therapeutic effects of the fused membrane formulations, oxidative damage models were established in BCEC and PC12 cells. The cells were separately incubated with LCP@miR, T-LCP@miR, EXO-LCP@miR, EXO-T-LCP@miR, EXOs, or T-LCP@NC for 48 h. Mitochondrial membrane potential was measured by flow cytometry to evaluate the in vitro antioxidant effects. Compared with that in the PBS and T-LCP@NC control groups, mitochondrial damage was significantly repaired in the LCP@miR, T-LCP@miR, EXO-LCP@miR, EXO-T-LCP@miR, and EXO groups, and the differences among these groups were not statistically significant (Figure 4B). This result differed slightly from the GFP transfection results and did not reflect the advantages of the fused membranes and targeted modifications. Possible reasons include the increased uptake and escape capacities of all the formulations in BCEC cells and the stronger reparative effect of miRNA-210 on endothelial cells.

In the oxidative damage model established in PC12 cells, all groups exhibited statistically significant differences in cellular damage compared to that in the PBS group (Figure 4C, *p* < 0.01). And the anti-oxidant activity of EXO-T-LCP@miR was enhanced by 26.5% compared with EXO-LCP (*p* = 0.0083) and by 18% compared with EXO (*p* = 0.0216). It has previously been shown that interventions targeting the CXCR4/SDF-1 axis facilitate the repair of oxidative damage in PC12 cells [23,24]. In the present study, the targeted formulation T-LCP, EXO formulation, and EXO-fused targeted formulation involved in CXCR4 receptor binding exhibited strong antioxidant effects, with the fused formulation exhibiting the strongest effect. This may be attributed to the superimposition of the effects of miRNA-210 and intervention targeting the CXCR4/SDF-1 axis.

### 3.6. In Vivo Brain Targeting of Fused Membrane Formulations in a Cerebral Ischemia–Reperfusion Animal Model

**Figure 5:**
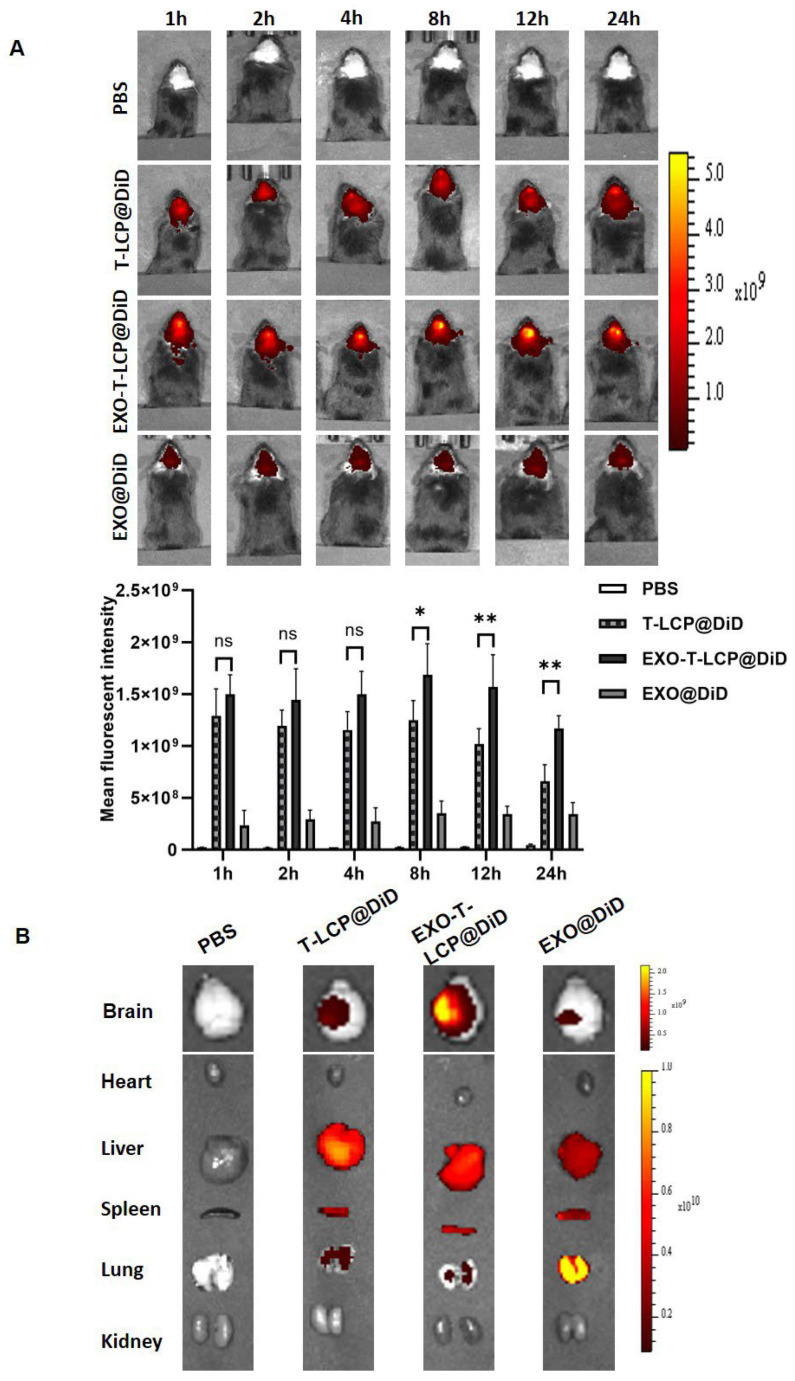
In vivo brain targeting of different nanoparticles in a cerebral ischemia–reperfusion animal model. (**A**) Real-time fluorescence images of MCAO mice with semi-quantitative results and tissues (**B**) acquired at different times after administration of different preparations. ^ns^ *p* > 0.05, * *p* < 0.05, ** *p* < 0.01.

Twelve and 24 h after the establishment of the MCAO model, intravenous injections of T-LCP@DiD, EXO-T-LCP@DiD, and EXO@DiD were administered, and in vivo targeting was examined using real-time fluorescence imaging. The fluorescence intensity in the brain 24 h after administration was obviously higher for both the T-LCP@DiD and EXO-T-LCP@DiD groups than for the EXO@DiD group (Figure 5A). EXO-T-LCP@DiD targeted the brain more effectively than the other treatments did and accumulated well in the brain even after 24 h. Within 4 h of administration, the fluorescence signal in the EXO-T-LCP@DiD group was clearly greater than that in the T-LCP@DiD group, although the difference was not significant (Figure 5A). Eight hours after administration, the fluorescence intensity of the EXO-T-LCP@DiD group continued to increase, with a significant difference in its accumulation in the brain compared to that of the T-LCP@DiD group (8 h: *p* = 0.0145, 12 h: *p* = 0.0013, 24 h: *p* = 0.0031). Therefore, both the targeted modification and EXOs possessed certain brain-targeting properties. However, targeting was significantly enhanced after fusion, clearly demonstrating that EXOs and the targeted modification could jointly enhance brain targeting.

Upon injection, each formulation reached various organs via blood circulation. Mice were subjected to cardiac perfusion 24 h after administration, and the brain, heart, liver, spleen, lungs, and kidneys were removed to visualize the distribution of the different formulations using a small-animal live imaging system. Imaging of the removed brain tissue (Figure 5B) revealed that fluorescent signals in both the targeted modification and EXO groups were clustered on the ischemic side of the brain, with the fused formulation group showing the strongest signals on the ischemic side. This result suggests that the CXCR4/SDF-1 axis contributed to more effective targeted delivery on the ischemic side of the brain, with the combined effects of the AMD3100 modification and EXO membrane-associated receptor expression improving lesion-targeted delivery. Therefore, the fused formulation combined the advantages of targeted modification and EXOs and achieved better targeted delivery to the ischemic side of the brain in the animal model.

T-LCP@DiD was primarily distributed in the liver and spleen tissues; the EXOs were mainly distributed in the lungs; and the fusion formulation was significantly less abundant in the lungs. The higher aggregation of the EXOs in the lungs may explain their lower aggregation on the ischemic side of the brain than that of the targeted formulation; it may also be due to the expression of multiple proteins in the EXO membrane. Higher accumulation of the fused formulation in the brain and lower accumulation in the lungs suggest that the fusion process led to the greater retention of properties of the AMD3100 modification, which were further enhanced by the EXOs.

### 3.7. Beneficial Effects of miRNA210-Loaded Fused Membrane Formulations on Rat Behavior with Ischemia–Reperfusion Injury

**Figure 6:**
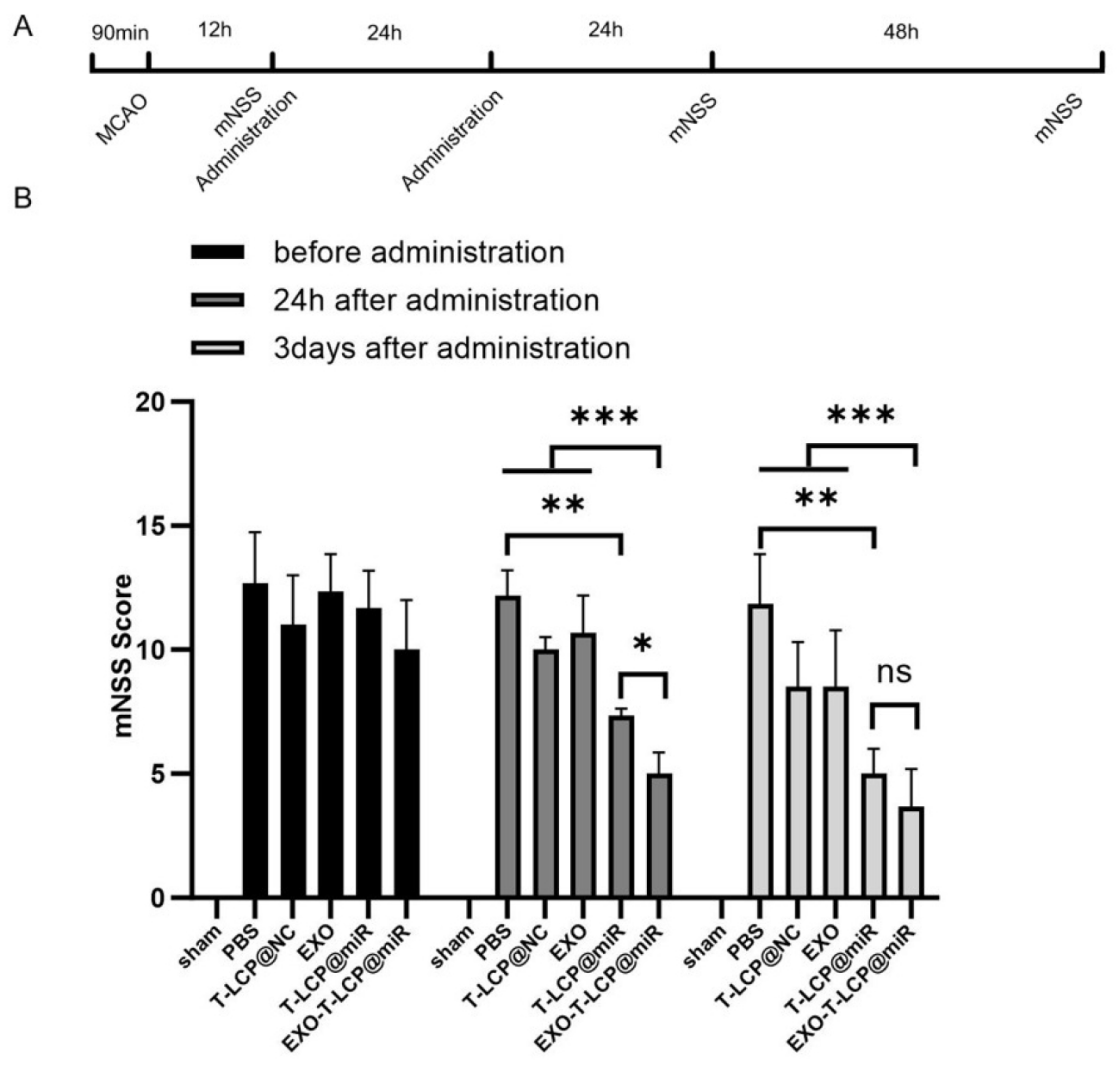
The beneficial effects of miRNA210-loaded nanoparticles on the behavior of rats with ischemia–reperfusion injury. (**A**): The experimental protocol for the behavioral evaluation; (**B**): The mNSS score of MCAO rats after administration of different preparations. ^ns^ *p* > 0.05, * *p* < 0.05, ** *p* < 0.01, *** *p* < 0.001.

Figure 6A shows the experimental protocol used to assess the effects of the miRNA-210-loaded formulations on the behavior of the animal models, and Figure 6B shows the mNSS scores for mice before treatment and 1st and 3rd days post-administration. The three groups showed no significant differences before administration (*p* > 0.05), indicating a consistent degree of modeling appropriate for pharmacodynamic assessment. One day after administration, both the T-LCP@miR (*p* = 0.0028) and EXO-T-LCP@miR (*p* = 0.0019) groups showed statistically significantly lower behavioral scores than the control group; however, the EXO group did not exhibit a therapeutic effect (*p* > 0.05). EXO-T-LCP@miR showed a stronger protection effect than T-LCP@miR (*p* = 0.0459). Three days after administration, EXO-T-LCP@miR and T-LCP@miR further improved the behavioral scores of the animals, which exhibited excellent recovery of neurological function. However, the two groups showed no statistically significant differences (*p* = 0.2745). The EXO group did not exhibit significant improvements in behavioral scores 3 days after administration (*p* > 0.05). This may be attributed to the fact that EXOs are secreted by cells and are of natural origin. Therefore, they possess diverse contents and do not specifically produce certain miRNAs with therapeutic effects. By contrast, T-LCP was artificially designed for the selective and efficient loading of therapeutic miRNA-210, which led to the maximization of its therapeutic effects. Hence, even though native EXOs have the potential to contribute to IS therapy, some undesirable features prevent their success in clinical applications, including a short half-life, poor targeting capacities, low concentrations at the target site, rapid clearance from the lesion region, and inefficient payload [13,25].

The targeting efficacy of EXO-T-LCP@miR was superior to that of T-LCP@miR; however, the two showed no significant differences in their therapeutic effects. EXOs possess a naturally occurring targeting ability and stronger biological activity, which led to the significant enhancement of targeting effects after fusion with T-LCP [13,26]. However, both EXO-T-LCP@miR and T-LCP@miR possessed the same CaP core with a similar ability to encapsulate miRNA-210. Although the fused formulation had a greater targeting capacity than that of the targeted formulation 24 h after administration, the difference between the two was not significant 3 days after administration. This suggests that after two cumulative administrations, both formulations could promote sustained miRNA-210 expression, compensating for the difference in the efficiency of targeted delivery by T-LCP@miR. Therefore, a significant therapeutic effect was observed after two administrations of the formulations over 3 days. In addition, the CXCR4/SDF-1 axis plays an important role in IS treatment [27,28]. Both T-LCP and EXO-T-LCP@miR utilized the CXCR4 receptor to achieve targeting, which may have contributed to the strong treatment efficacy of both formulations. Nonetheless, fused formulations in targeted delivery hold great potential for further development.

## 4. Conclusions

In the present study, the unique asymmetric membrane structure of LCP was utilized to prepare a novel fused-membrane NP via the fusion of an outer monolayer with an EXO membrane. The NP drug delivery system possessed a high-density, positively charged CaP core, which enabled the efficient loading of the gene drug miRNA-210. The combination of the AMD3100 targeting moiety modification and EXO membrane in the outer membrane structure enhanced targeting towards the ischemic brain region. In addition, the lysosomal escape characteristics of the CaP core were utilized to enhance the intracellular stability of the gene drug, allowing miRNA-210 to exert a prominent therapeutic effect in an animal model of cerebral ischemia. Comparative analyses of targeted nanoscale formulations, EXOs, and fused formulations revealed that the targeted delivery system designed based on the CXCR4/SDF-1 axis possessed significant targeted delivery advantages, with the fusion of the EXO biomembrane further enhancing the targeting capacity. The results presented herein demonstrate that the fusion of artificially designed NPs with EXOs of natural origin is feasible and combines the advantages of both to achieve more prominent targeted delivery effects. MiRNA210-based gene therapy was determined to be effective and provides a strategy for good therapeutic effects in delivery systems with different targeting efficiencies. Therefore, the present study provides a novel and highly promising approach for the future design of nanoscale formulations and gene therapy vectors.

## Figures and Tables

**Table 1:** Mean diameter and zeta potential of membrane fusion nanoparticles.

EXO:LCP	Size (mm)	PDI	Zeta Potential (mV)
LCP	97.32 ± 2.26	0.291 ± 0.01	14.4 ± 0.63
1:9	75.15 ± 0.37	0.252 ± 0.01	−7.55 ± 2.13
1:4	87.32 ± 2.57	0.268 ± 0.01	−8.30 ± 1.40
1:1	90.79 ± 1.31	0.293 ± 0.02	−14.3 ± 0.30

## Data Availability

All data included in this study are available upon request from the corresponding author.

## Abbreviations

The following abbreviations are used in this manuscript:

BMSC	bone marrow stromal cell
CaP	calcium phosphate
CCA	common carotid artery
DLS	dynamic light scattering
EXO	exosome
ECA	external carotid artery
FCS	fluorescence correlation spectroscopy
ICA	internal carotid artery
IS	ischemic stroke
LCP	lipid calcium phosphate
NP	nanoparticle
miRNA	microRNA
NTA	nanoparticle tracking analysis
PBS	phosphate-buffered saline
PDI	polydispersity index
TEM	transmission electron microscope
T-LCP	AMD3100-modified targeted LCP
